# A Simplest Method to Enhance the Benefits of Internal Thoracic Artery Distal Occlusion

**DOI:** 10.21470/1678-9741-2020-0308

**Published:** 2021

**Authors:** Yoandy López-de la Cruz, Yolepsis Fidel Quintero-Fleites, Manuel Nafeh Abi-Rezk

**Affiliations:** 1 Department of Cardiovascular Surgery, Santa Clara Cardiac Center, Santa Clara, Villa Clara, Cuba.; 2 Department of Cardiovascular Surgery, Hermanos Ameijeiras Hospital, Havana Center, Havana, Cuba.

**Keywords:** Mammary Arteries, Hydrostatic Pressure, Dissection, Cardiovascular System, Hemorrhage

## Abstract

A common element of internal thoracic artery harvesting techniques is a distal vascular clamp placement at the end of the procedure, not only to avoid bleeding, but also to increase the internal hydrostatic pressure, diameter and flow.

The logic indicates that the placement of this clamp at the beginning of the dissection will allow the artery to benefit earlier from these advantages.

After more than five years of experience, we present a modification in the classical technique of skeletonized harvesting of the internal thoracic artery, consisting of artery distal occlusion at the beginning of the procedure. Some of its advantages are discussed.

**Table:** 

Abbreviations, acronyms & symbols	
**ITA**	**= Internal thoracic artery**

## INTRODUCTION

Obtaining high flow in the internal thoracic artery (ITA) has been a concern of cardiac surgeons since the dawn of the era of direct myocardial revascularization. This topic has become more important in the last two decades, as the strategy of revascularization of the anterolateral heart wall with bilateral internal thoracic arteries has gained wide acceptance.

A growing body of evidence shows that the flow of the arterial graft is the determining factor of its quality and patency in any period analyzed^[1,2]^. Several factors condition it, but the blood flow is one of the few that the surgeon can modify at the time of surgery.

Skeletonized artery harvesting, administration of various vasodilators or antispasmodics and the development of safer cauterization technologies have been the main methods used by surgeons to increase the diameter and flow of arterial conduits. On the other hand, at some point in the historical evolution of the ITA dissection procedures, surgeons realized that the distal occlusion of the artery at the end of its harvesting caused an increase in its diameter and flow. Little has been written about that physiological response^[3,4]^.

For the past 5 years, we have completely dissected the ITA with its distal end occluded, without the need for patient heparinization. Probably no other group does ITA harvesting that way, so we want to share the benefits of this new method with a greater number of cardiothoracic surgeons.

## TECHNIQUE

**Fig. 1:**
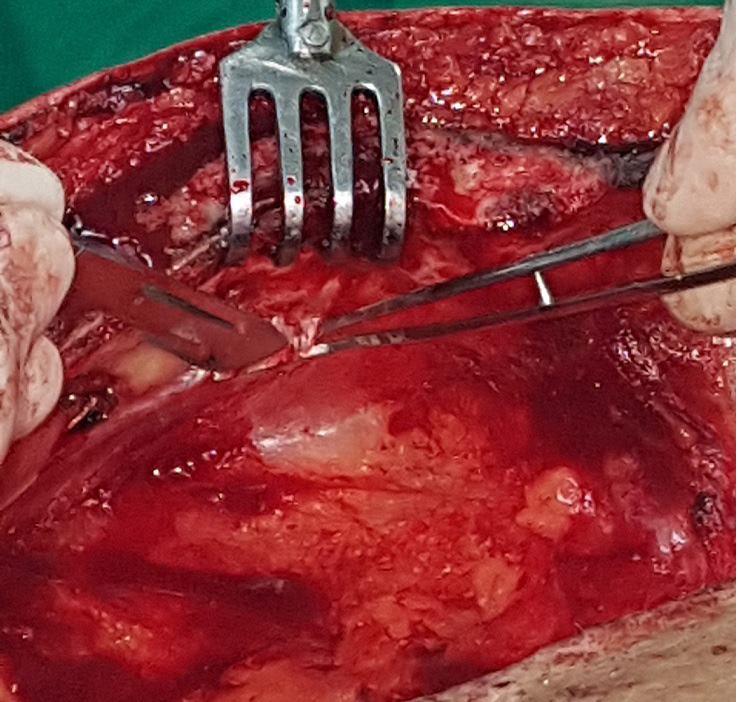
Opening of the endothoracic fascia covering the distal segment of the left ITA. The use of a scalpel facilitates the maneuver.

**Fig. 2:**
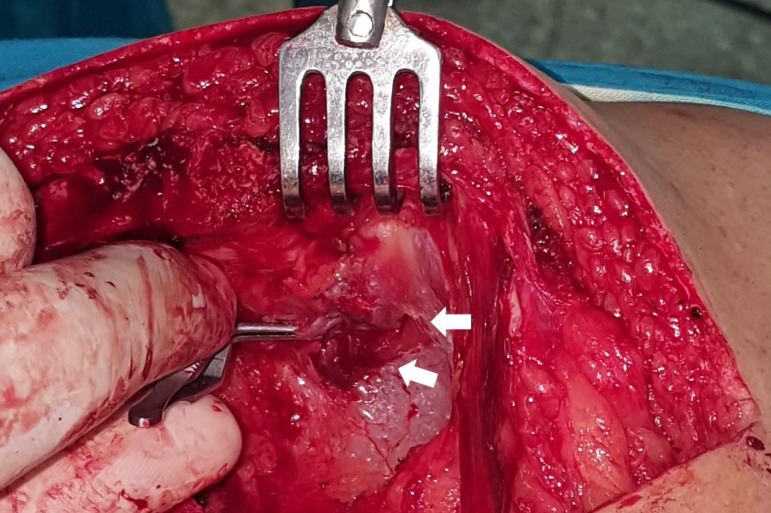
Detail of the left ITA dissection. The fibers of the transversus thoracis muscle that covered the distal segment of the artery were cut without bleeding (white arrows).

First, the endothoracic fascia or transverse thoracic muscle fibers that cover the distal segment of the ITA are opened. Although apparently other groups do not use it, we have observed that the opening of these structures with a scalpel is very easy and fast, with practically no risk of injury to the artery or muscle bleeding (Figures 1 and 2). The use of electrocautery will take longer and there will always be a risk of thermal injury to other structures, even at low power.

**Fig. 3:**
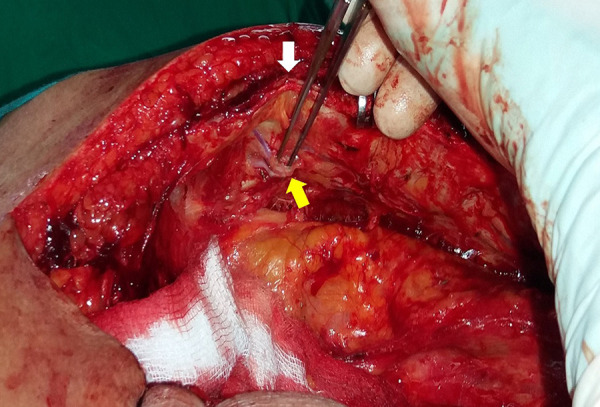
Detail of the right ITA distal dissection. Observe the bifurcation in two terminal branches (yellow arrow) at the level of the union of the sternum body with the xiphoid process (white arrow).

The presence of two or three branches practically the same diameter as the previous segment of the vessel, in the artery section that coincides with the union of the sternum body and its xiphoid process, are anatomical clues to adequately identify the end of the ITA. In most of the population, the ITA ends at the 6^th^ intercostal space, a point that coincides with the aforementioned articulation (Figure 3). 

**Fig. 4:**
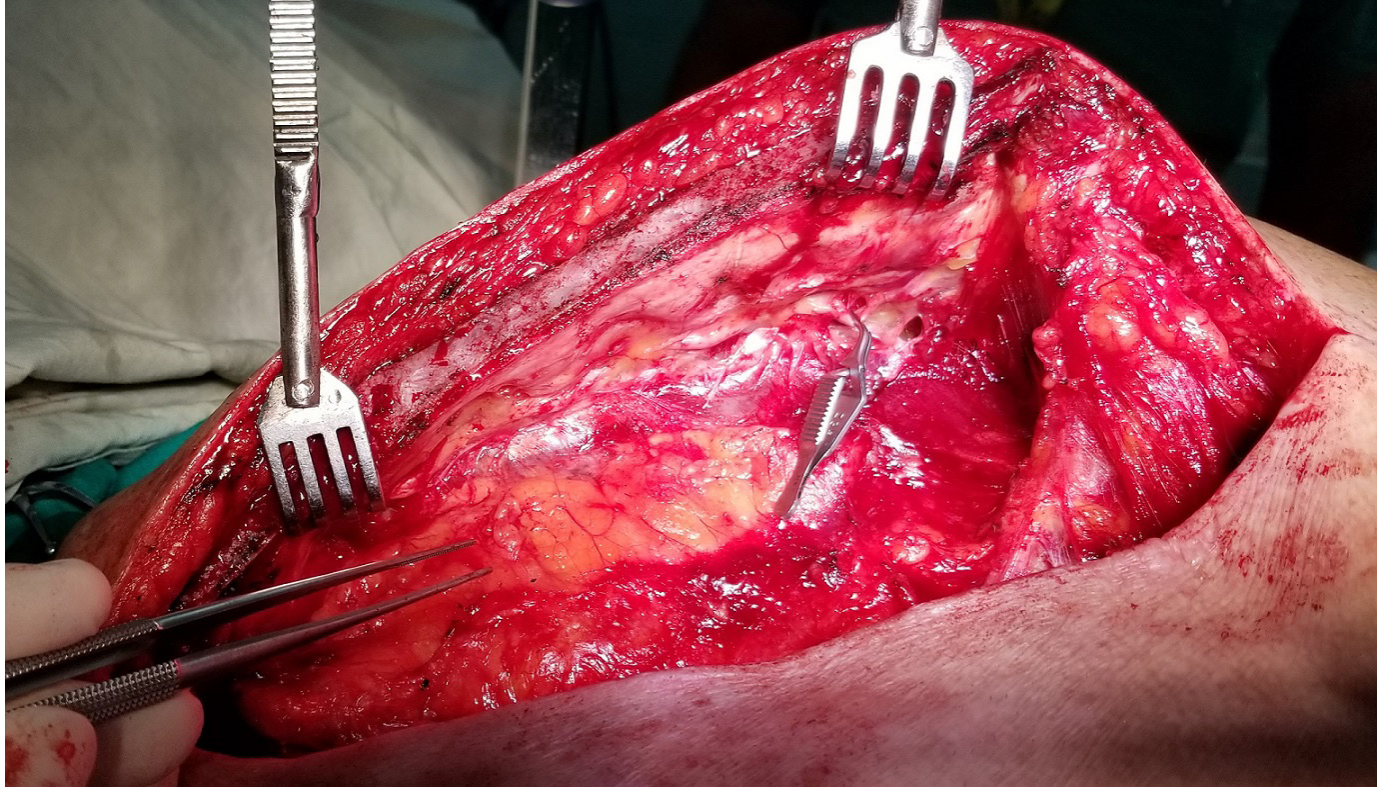
Pronounced elevation of the distal segment of the sternum will favor the dissection of the ITA bifurcation without risk of skeletal injury.

The use of a retractor that allows a pronounced elevation of the distal third of the sternum will notably facilitate the identification of the ITA terminal branches (Figure 4). In our practice, there was no sternum fractures or rib disarticulation caused by a marked retraction of that bone segment.

Finally, without the need for prior heparin administration, a bulldog clamp is placed before the bifurcation (Figures 2 and 4), and dissection and control of the rest of the branches is initiated.

## DISCUSSION

Recent studies^[5,6]^ have shown that when all the lateral branches of the ITA are occluded during their harvesting, their native (physiological) flow decreases; therefore, there appears to be no benefit to the strategy of keeping ITA distally patent during dissection.

On the other hand, after dissection, with the ITA clamped for a period, the initially low arterial flow will increase considerably. Therefore, the ITA distal occlusion at the beginning of its dissection allowed an earlier onset of its dilation and increased flow^[7]^.

A main determinant of the anterograde flow of the ITA is the reverse bloodstream (flow competence) that reaches the distal end of the vessel, due to the connections of its final branches (superior epigastric and musculophrenic artery) with other arterial systems (distal segments of the thoracic aorta, external iliac artery, etc.). For this reason, the free flow of the ITA (against zero resistance) will always be greater than when connected to the circulation, either in its natural location or anastomosed to a coronary artery. 

**Fig. 5:**
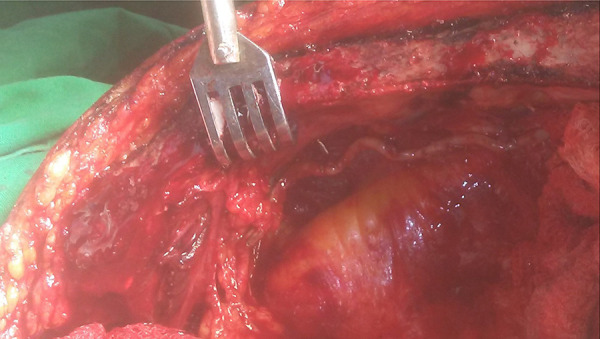
Considerable dilation of the middle segment of the left ITA during the harvesting with its distal end occluded.

Early occlusion of the artery causes an abolition of flow competition in both directions, so its antegrade and retrograde flows will begin to increase. The increased flows will cause a greater production of nitric oxide by the enzyme endothelial nitric oxide synthetase, which will dilate the artery and further increase the flow, initiating a very favorable vicious circle for the hemoduct quality (Figure 5).

In a recently completed randomized control trial (unpublished data), we found that the free flow of ITA (post-harvesting) was significantly higher when the artery was occluded distally at the beginning of its dissection (83±24 *vs*. 44±15 mL/min.; *P*<0.001). In almost half of the patients, flows higher than 100 mL/min were obtained. In no case heparinization was necessary before the placement of the clamp, since it has been known for decades that there is no risk of thrombosis when the ITA is distally occluded.

At the same time, the distal occlusion of the ITA during harvesting will cause an intentional redistribution of blood flow to the sternum and other mediastinal organs, either directly (ITA's sternal branches) or by feeding collateral circulation channels (connections of the pericardiophrenic artery with epicardial branches of the coronary arteries)^[8]^. One of the principles of arterial circulation states that a vessel occlusion will cause opening of pre-existing channels, due to the increase in local intravascular pressure. This happens especially in the ITA, due to its known significant potential for plasticity in the presence of an obstruction to the anterograde flow. The pressure elevation inside the ITA, caused by distal clamp placement, will lead to an increase in blood flow to the heart through sources of non-coronary myocardial collateral circulation.

This new myocardial blood flow will be favored by the presence of extensive networks of coronary collateral circulation in patients who usually have had myocardial ischemia for many years. Furthermore, this collateral irrigation will be both anterograde and retrograde, thanks to the large number of anastomoses that establish the pericardiophrenic artery and other final branches of the ITA at the level of the diaphragm^[9]^.

On the other hand, the intentional redistribution of increased blood flow to the sternum during the ITA harvesting is another important advantage obtained from the artery distal occlusion. Since ITA is the most important source of sternum blood supply, a certain amount of the antibiotic (antibioprophylaxis) that will protect the bone from future infectious complications will be distributed far from the operative field when the vessel is distally patent. Nevertheless, the ITA distal occlusion as close as possible to the antibioprophylaxis administration will allow the sternum impregnation with an antimicrobial amount, even higher than would occur under normal conditions. In the near future, bilateral ITA distal occlusion just after sternotomy, or even preoperatively by means of catheter-mediated procedures, besides favoring myocardial irrigation coming from extracardiac sources^[10]^, could greatly reduce the incidence of sternal healing complications when both internal thoracic arteries are used.

## CONCLUSION

Internal thoracic artery harvesting with its occluded distal end offers significant benefits related to the quality of the graft and protection of the mediastinal organs against future infectious complications. However, these physiological advantages have apparently not been perceived by cardiac surgeons, as ITAs are currently occluded only at the end of their preparation.

The high production of heparin sulfate in the endothelium of the ITA allows clamping without the need for systemic heparinization of the patient, which reduces the risk of bleeding or conduit injury during handling. Our study demonstrated that the ITA can be occluded for a long time without risk of thrombosis or endothelial injury. Furthermore, the postoperative outcomes of the patients were satisfactory.

**Table:** 

Authors' roles & responsibilities	
YLC	Substantial contributions to the conception or design of the work; or the acquisition, analysis, or interpretation of data for the work; drafting the work or revising it critically for important intellectual content; final approval of the version to be published
YFQF	Substantial contributions to the conception or design of the work; or the acquisition, analysis, or interpretation of data; final approval of the version to be published
MNAR	Final approval of the version to be published
